# Surface Plasmon Enhanced Light Scattering Biosensing: Size Dependence on the Gold Nanoparticle Tag

**DOI:** 10.3390/s19020323

**Published:** 2019-01-15

**Authors:** Chih-Tsung Yang, Yi Xu, Mohammad Pourhassan-Moghaddam, Duy Phu Tran, Lin Wu, Xin Zhou, Benjamin Thierry

**Affiliations:** 1 Future Industries Institute and ARC Centre of Excellence in Convergent Bio and Nano Science and Technology, University of South Australia, Mawson Lakes Campus, Mawson Lakes 5095, Australia; chih-tsung.yang@unisa.edu.au (C.-T.Y.); duy.tran@unisa.edu.au (D.P.T.); 2 Electronics and Photonics Department, Institute of High Performance Computing, Agency for Science, Technology, and Research (A*STAR), Singapore 138632, Singapore; yi_xu@mymail.sutd.edu.sg (Y.X.); wul@ihpc.a-star.edu.sg (L.W.); 3 SUTD-MIT International Design Center & Science and Math Cluster, Singapore University of Technology and Design, Singapore 487372, Singapore; 4 School of Biomedical Engineering, University of Technology Sydney, Sydney 2007, Australia; pourhassanmo@gmail.com; 5 Institute for Biomedical Materials and Devices (IBMD), Faculty of Science, University of Technology Sydney, Sydney, NSW 2007, Australia; 6 Institute of Comparative Medicine, Yangzhou University, Yangzhou 225009, China; zhou_xin@126.com

**Keywords:** surface plasmon resonance, surface plasmon enhanced light scattering, gold nanoparticles, signal amplification, gold enhancement

## Abstract

Surface plasmon enhanced light scattering (SP-LS) is a powerful new sensing SPR modality that yields excellent sensitivity in sandwich immunoassay using spherical gold nanoparticle (AuNP) tags. Towards further improving the performance of SP-LS, we systematically investigated the AuNP size effect. Simulation results indicated an AuNP size-dependent scattered power, and predicted the optimized AuNPs sizes (i.e., 100 and 130 nm) that afford extremely high signal enhancement in SP-LS. The maximum scattered power from a 130 nm AuNP is about 1700-fold higher than that obtained from a 17 nm AuNP. Experimentally, a bio-conjugation protocol was developed by coating the AuNPs with mixture of low and high molecular weight PEG molecules. Optimal IgG antibody bioconjugation conditions were identified using physicochemical characterization and a model dot-blot assay. Aggregation prevented the use of the larger AuNPs in SP-LS experiments. As predicted by simulation, AuNPs with diameters of 50 and 64 nm yielded significantly higher SP-LS signal enhancement in comparison to the smaller particles. Finally, we demonstrated the feasibility of a two-step SP-LS protocol based on a gold enhancement step, aimed at enlarging 36 nm AuNPs tags. This study provides a blue-print for the further development of SP-LS biosensing and its translation in the bioanalytical field.

## 1. Introduction

Surface plasmon resonance (SPR) has matured into one of the most powerful and versatile bioanalytical techniques. It has been widely employed to monitor biomolecular binding events including cells [1,2,3], proteins [4,5] and nucleic acids [6] and more generally for the development of medical diagnostic technology [7]. Signal amplification tags are commonly employed to enable the detection with conventional SPR set-up of analytes of either low molecular weight or at ultra-low concentrations. Along with SPR amplification strategies based on fluorescent dyes [8,9] and enzymatic reactions [10,11], gold nanoparticles (AuNPs) [12] have been extensively used due to their unique optical property, stability, and ease of preparation. In the standard sandwich sensing configuration, AuNP tags modified to bind to the molecular target are used to drastically increase the refractometric signal associated to the capture of the target on the SPR sensor.

We have previously reported the novel concept of surface plasmon enhanced light scattering (SP-LS) biosensing [13]. In this paradigm, the scattered light generated by AuNP tags is induced by the excitation of propagating surface plasmons (PSPs) at the AuNP/water interface, which is transmitted to optical power scattered by AuNPs. The enhanced sensitivity is associated to the strong electromagnetic field enhancement of the AuNPs, which is converted into strong scattering signals and allows the improved detection of the target analytes. SP-LS with spherical AuNP tags with diameters of 36 nm resulted in approximately three orders of magnitude improvement in sensitivity as compared with that of conventional refractometric SPR measurements for the detection of cardiac troponin-I and miRNA [13,14]. However, while the AuNP size effect has been thoroughly investigated for refractometric SPR sensing [15], as well as for SPR based on phase measurement [16,17], this knowledge is missing for SP-LS sensing. Scattered light intensity is highly dependent on particle size according to the Rayleigh-Gans-Debye approximation [18,19,20], and there is therefore significant scope to further increase the performance of SP-LS biosensing by elucidating the effect of the size of the AuNP tags. To this end, we conducted both simulation and experimental studies designed to fully investigate the size dependence of AuNP tags. In addition, we also optimized a protocol for the bioconjugation of the AuNP molecular tags. Previous studies have indeed shown that a careful balance is needed between optimizing the signal enhancement associated to the binding of the AuNP tags onto the sensor surface and maximizing the overall binding of the AuNP tags to the target captured on the surface [21]. Finally, building on the simulation data, we also demonstrate the feasibility of a two-steps SP-LS biosensing protocol based on a gold-enhancement method aimed at increasing the size, and therefore the signal of the AuNP tags.

## 2. Materials and Methods

### 2.1. Materials

Chloroauric acid (HAuCl_4_·3H_2_O), sodium citrate, hydroquinone, Tween 20, *N*-hydroxy-succinimide (NHS), *N*-(3-dimethylaminopropyl)-*N*-ethylcarbodiimide hydrochloride (EDC), sodium chloride, hydroquinone and phosphate buffer saline (PBS) were purchased from Sigma Aldrich (Castle Hill NSW, Australia). HS-(PEG)_7_-COOH (MW 456.8 Da) was acquired from Polypure (Oslo, Norway) and HS-(PEG)_x_-OMe (MW 2000 and 5000 Da) was obtained from Rapp Polymere (Tübingen, Germany). Mouse anti-goat IgG antibody (cat. No#. SAB3700264-1MG), Goat anti-mouse IgG antibody (cat. No#. SAB3701071-2MG), bovine serum albumin (BSA), horseradish peroxidase (HRP) enzyme were purchased from Sigma-Aldrich. Cellulose nitrate strip (pore size 0.45 µm) was purchased from Sartorius Stedim Biotech GmbH (Goettingen, Germany). PBST was prepared by addition of 0.1% tween-20 (Sigma-Aldrich) in PBS. TMB and H_2_O_2_ substrate were supplied by Zhengzhou Humanwell Biocell Biotechnology (Zhengzhou, China).

### 2.2. Synthesis of Au Seeds and AuNPs

Au seeds of ~17 nm size were synthesized by the standard citrate reduction method [22]. AuNPs of different sizes were subsequently synthesized by mixing specific volumes of the as-synthesized Au seeds, AuHCl_4_ (1%), sodium citrate (1%) and hydroquinone (0.03 M) at room temperature. The size distribution of the AuNPs was measured using a Zetasizer Nano ZS equipped with a 633 nm He-Ne laser from Malvern Instruments (Malvern, UK). Fluctuations in the intensity of scattered light (at 90° to the incident) were analyzed through the use of first-order and second-order autocorrelation functions. The Z-Average size and polydispersity index were obtained using the manufacturer’s software based on the cumulants method. UV-Vis absorption spectra were acquired on a Cary 5 UV-Vis-NIR spectrophotometer (Varian, CA, USA).

### 2.3. Growth of AuNPs with Gold Enhancement Reagent

As-prepared AuNPs were enlarged following a gold enhancement (GE) protocol [23]. The growth reactions were performed in solution as well as directly on the SPR chip to increase the size of surface-bound AuNPs. Briefly, fresh GE reagent was prepared immediately before the growth reaction by mixing HAuCl_4_ with NH_2_OH at the volume ratio 6 to 1. In the solution phase, 1 volume of the as-prepared AuNPs was mixed with 1 volume of the GE reagent and the mixture was incubated at room temperature at three different time intervals. UV-Vis was used for monitoring the localized SPR (LSPR) shift of the AuNPs before and after the growth reaction. The size and morphology of the enlarged AuNPs were studied by SEM. In addition, the growth reactions were performed on the surface of SPR chip by exposing the chip surface-bound AuNPs to the GE reagent for 5 min.

### 2.4. AuNP Conjugation with IgG

As-synthesized AuNPs were functionalized with goat anti-mouse IgG using EDC/NHS chemistry. Briefly, AuNPs were washed by centrifugation at 16,000 rpm for 10 min. A fresh 4:1 (*v/v*) ratio of HS-(PEG)_7_-COOH (2.18 mM) to HS-(PEG)_x_-OMe (2.18 mM) was added to the washed AuNPs and the mixture was incubated overnight at room temperature. The resulting PEGylated AuNPs were washed twice by centrifugation at 16,000 rpm for 10 min. The second pellet was reacted with a fresh solution of EDC/NHS (0.4 M/0.1 M) for 10 min at room temperature to activate the carboxylic groups of the PEGylated AuNPs. The excess EDC/NHS was washed off by centrifugation. Immediately, mouse anti-goat IgG (1 mg/mL) was incubated with the activated AuNPs for 3 h at room temperature. The excess reagents were removed from the bio-conjugated AuNPs by centrifugation of the mixture at 4 °C, and the samples were resuspended in PBST containing 1% BSA and stored at 4 °C until use.

### 2.5. Confirmation of Bioconjugation with Dot Blot Assay

A dot blot assay was used to investigate the immune-binding efficiency of the prepared antibody-AuNP conjugate in recognizing mouse anti-goat IgG used as a model target antigen. Briefly, three different concentrations of mouse anti-goat IgG (1, 0.1, 0.01 mg/mL) in PBS were spotted on cellulose nitrate strips. Goat anti-mouse IgG (1 mg/mL) was spotted as control. The blotted samples were left for 15 min at room temperature to air-dry. Then, the strip was immersed in 5% BSA for 30 min at room temperature as a blocking step. The strip was incubated with the antibody-AuNP (17 nm) conjugate (OD = 0.2) for 2 h at room temperature. The excess AuNPs were washed off twice with MQ water containing 0.05% Tween-20. The color change was observed by the naked eyes.

### 2.6. SPR Sensor Preparation and Optical Setup

48 nm of gold film on top of 2 nm of Cr film was sputtered on LaSFN9 glass to prepare SPR sensors as previously described [24]. The detailed optical setup for SP-LS is depicted in Figure S1. Briefly, the flow-cell made of PDMS spacer with a volume of 25 μL was pressed against the antibody functionalized sensor and a quartz lid connected with the tubing (inner diameter = 0.13 mm, Tygon R3607) through the inlet and outlet linked to a peristaltic pump at the flow rate of 20 μL/min for sample circulation. The scattered light emitted from the sensor surface was collected through the flow-cell by a lens (numerical aperture NA = 0.3), passed through a ND filter, and its intensity was detected by a photomultiplier tube (PMT). The measurement of refractometric scheme (angle of incidence vs reflectance) was collected by photodiode and the measurement of scattered light scheme (angle of incidence vs scatted light intensity) was monitored by PMT. Notably, these two schemes can be measured at the same time through the Wasplas software developed at the Max Planck Institute of Polymer Research (Mainz, Germany).

### 2.7. Simulations

The simulation of the SPR-LSPR coupling system was conducted with the three-dimensional (3D) finite element method using COMSOL Multiphysics 5.3 with the RF Module. The computational domain is a unit cell consisting of one AuNP surrounded by water sitting on a multilayered SPR substrate containing a prism LaSFN9, 2 nm-thick Cr film, 48 nm-thick Au film, and a 10 nm-thick dielectric coating. The 10 nm spacing was chosen based on the approximated distance between the Au film and AuNP tags for the anti-IgG model system used in the experimental study. The lateral dimension of the unit cell was set as the pitch p, i.e., the center-to-center distance between AuNPs. Periodic boundary conditions were employed at the sides of the unit cell, while water and LaSFN9 perfectly matched layers were applied at the top and bottom of the analyzed structure. In the simulations, the refractive index of the 48 nm-thick Au film was taken as 0.18 + 3.5*i* under the illumination of a 632.8 nm excitation light, and 0.6 + 2.25*i* for the AuNPs. The refractive index for the chromium (Cr) film is 3.14 + 3.31*i*, and it is 1.333, 1.845, and 1.46 for water, prism LaSFN9, and the dielectric coating, respectively.

A TM-polarized excitation light source with the fixed wavelength of 632.8 nm was introduced in the LaSFN9 domain. The incident light propagates from the prism LaSFN9 into the Au film-AuNPs coupling system, and was absorbed, reflected, or transmitted on striking the coupling system. The absorbed power was calculated by the volume integration of the resistive heating in the metallic nanostructure (i.e., Cr film, Au film, and AuNPs). The reflected (or transmitted) power was calculated by the surface integration of far-field power flow in the prism LaSFN9 (or water) domain. The incident angle of the TM-polarized light was varied to match the SPR condition.

After solving the 3D Maxwell’s equations, the absorption, reflection, and transmission as a function of the incident angle was obtained. The scattered power from AuNPs was computed through the difference of the transmitted power with and without AuNPs, and normalizing it with the area of the simulated unit cell. In addition, the electric field distribution at the resonant angle was obtained from the COMSOL simulations.

## 3. Results and Discussion

### 3.1. Theoretical Modeling

**Figure 1:**
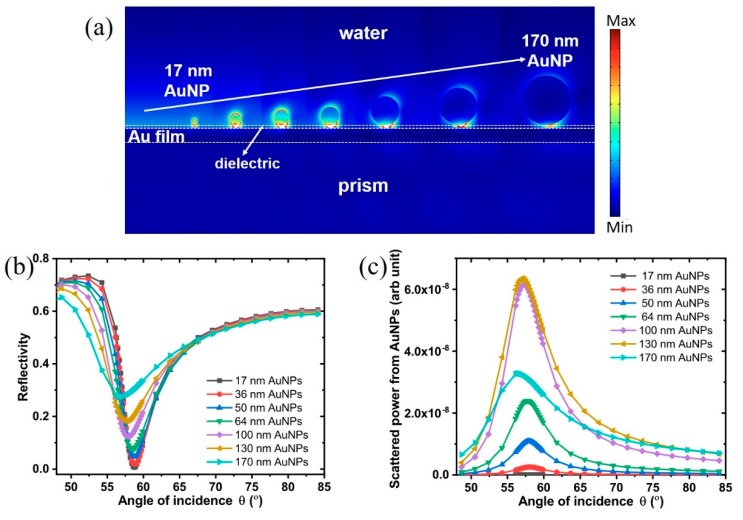
(**a**) Simulation of the field enhancement distribution on the SPR sensor surface for AuNPs with diameters ranging from 17 to 170 nm. Simulated angular SPR spectra (**b**) and scattered power (**c**) as a function of the incident angle AuNP probes with diameters ranging from 17 to 170 nm. Configuration for simulation: dielectric layer thickness of 10 nm; pitch between AuNPs at p = 800 nm.

In the SP-LS sensing mode, SPs are excited with the Kretchmann configuration [25], when the following condition is fulfilled:

(1) $$k_{x}=k_{sp}$$

where $k_{x}=k_{0}n_{LaSFN9}\sin\theta_{0}$ is the x-component of the wavevector of incident light that is parallel to the Au film, and $k_{sp}$ is the wavevector of SP oscillations. $k_{0}=2\pi /\lambda$ is the wavevector of the incident light (with the wavelength λ=632.8 nm) in free space, and $n_{LaSFN9}=1.845$ is the refractive index of the prism LaSFN9. $\theta_{0}=52.4{}^{\circ}$ is the angle of light at the interface between prism LaSFN9 and Au film, while the incident angle at the air/prism interface is $\theta_{inc}=58.75{}^{\circ}$. Therefore, the excited SP possesses the wavelength of $\lambda_{SP}=433$ nm calculated from Equation (1). The excited PSP along the Au film then interacts with the AuNPs, and excites the localized SPs (LSPs) of the AuNPs, resulting in localized electromagnetic field around the AuNPs, as shown in Figure 1a. The AuNP size-dependent reflectivity-incident angle curves are plotted in Figure 1b. The minimum reflectivity increases with the AuNP size due to the increasing plasmon damping, which has been experimentally reported [25]. For the angular resonant dips, 17 nm-, 36 nm-, and 50 nm-sized AuNPs possess the same resonance angle (58.75°) as that of AuNPs-devoid case (see Figure 1b). The invariable resonance angle after the adsorption of AuNPs can be attributed to the small size of AuNPs and the relatively low particle surface coverage (p = 800 nm) has negligible effect on the plasmon shifts. With relatively large AuNPs (64 nm, 100 nm, 130 nm, and 170 nm), the resonance angle decreases with the AuNP size, as shown in Figure 1b. The negative angular shift of the resonant dip has been previously reported by Uchiho et al. for the adsorption of 150 nm AuNPs [26].

The AuNP size-dependent scattered power as a function of the incident angle is shown in Figure 1c. The power scattered from AuNPs increases with the AuNP size from 17 nm to 130 nm. 17 nm AuNPs exhibited negligible scattered power as compared to the larger AuNPs. The maximum power (i.e., the peak) scattered from 130 nm AuNPs is found to be about 1700-fold higher than that from 17 nm AuNPs. This result confirms that larger AuNPs provide higher signal amplification in the SP-LS sensing scheme. However, the maximum scatter power for 170 nm AuNPs is about half of that obtained for 100 nm and 130 nm AuNPs, demonstrating the existence of an optimized size. This behavior is presumably due to the combined effect of the PSP-LSP coupling and the scattering effect of AuNPs. It has been demonstrated that with the adsorption of AuNPs on the SPR sensor surface, there is significant variations in the extinction and scattering spectrum of AuNPs [27,28]. The wavelength of LSP for the 17 nm-, 36 nm-, 50 nm-, 64 nm-, 100 nm-, 130 nm-, and 170 nm-sized isolated AuNPs (i.e., not coupled with PSP) are 523 nm, 526 nm, 530 nm, 537 nm, 570 nm, 605 nm, and 683 nm, respectively (see the normalized extinction efficiency Qext in Figure S2a, supporting information). Therefore, the efficiency of PSP-LSP coupling decreases with the AuNP size due to the increasing gap between the wavelengths of LSP and SP ($\lambda_{SP}=433$ nm). The scattering efficiencies Q_sca_ for isolated AuNPs with different sizes were compared in Figure S2b, which reveals that smaller AuNPs scatter less power at the wavelength of SP $\lambda_{SP}$. The combination of these two effects results in the existence of an optimal AuNP size, 130 nm in this case, for the scattered power in the SP-LS sensing scheme.

### 3.2. Optimization of Mixed PEG Coatings

**Figure 2:**
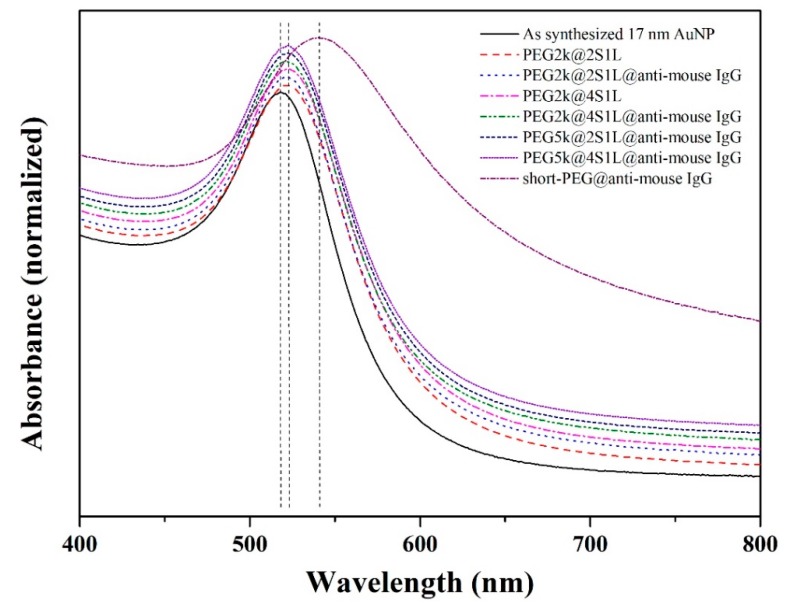
UV-Vis spectra of as synthesized AuNPs (17 nm) and AuNPs bioconjugated with an anti-mouse IgG via various poly(ethylene glycol) linkers.

Simulation demonstrated that large AuNPs are preferable as they induce greater scattered signals. AuNPs with various sizes were therefore synthesized to experimentally investigate the AuNP size-dependence in the SP-LS scheme. AuNPs were first PEGylated prior to the bioconjugation of antibodies. However, significant aggregation occurred for larger AuNPs (> 64 nm) during the antibody modification step, which prevented their utilization in SP-LS measurements. The optimization of PEG coatings is not trivial as the polymeric biointerface plays a crucial role in both maintaining the colloidal stability of the samples as well as controlling the immuno-binding efficiency to biological targets [29,30]. The as-synthesized AuNPs were functionalized with the heterobifunctional PEG molecules. Building on our previous study for Au nanorods [21], different molar ratios of high molecular weight PEG (MW 2000 Da and MW 5000 Da) and low molecular weight PEG (MW 458.6 Da) were employed to optimize the colloidal stability as well as immuno-binding efficiency to molecular targets bound onto solid substrates. For instance, AuNPs functionalized with low molecular weight PEG and high molecular weight PEG at the molar ratio of 2 to 1 is denoted as PEG2k@2S1L. The carboxylate end group of the PEG molecules was activated with standard carbodiimide chemistry to conjugate goat anti-mouse IgG. Goat anti-mouse and mouse anti-goat IgG were used in this study as a model immunoassay [31,32] to investigate the size-dependence of SP-LS. As shown in the UV-Vis spectra (Figure 2), an 8 nm blue shift of the absorbance peak was measured after PEGylation in the case of 17 nm AuNPs; there was no significant shift in the absorbance peak after the conjugation of antibodies. In order to further validate the successful modification of antibody on AuNP surface, DLS was used to characterize the increment of particle size. We measured increases of the hydrodynamic thickness (Table 1) for PEG2k modified AuNPs of ~4 nm and ~19 nm, respectively, for PEG2k@S2L1 and PEG2k@S4L1, suggesting that more antibodies can be conjugated on the AuNPs with more activated sites (i.e., higher ratio of LMW PEG). However, there was negligible increase in the particle size in the case of PEG5k, which might be attributed to the fact that longer PEG chain sterically limits the bioconjugation.

### 3.3. Confirmation of the Immuno-Binding Efficiency Based on Dot Blot Assay

**Figure 3:**
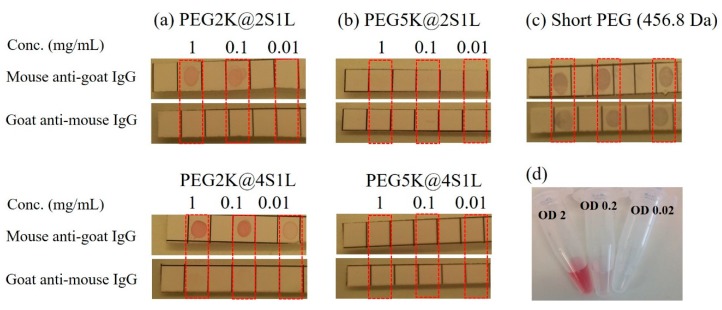
Dot-blot strips analysis of immune-binding efficiency for PEGylated AuNPs (17 nm) coated with various ratios of short to long PEG molecules. Photographic images of the dot-blot strips showing the specific (Mousse anti-goat IgG strips) and non-specific (Goat anti-mousse IgG strips) adsorption of goat anti-mouse IgG bioconjugated AuNP probes with various PEG coatings: (**a**) PEG2K@2S1L and PEG2K@4S1L; (**b**) PEG5K@2S1L and PEG5K4S1L; (**c**) only short PEG. (**d**) Photographic image of the AuNP solutions at the tested concentrations.

An IgG immuno-dot blot assay was employed to further confirm antibody functionality after conjugation onto AuNPs. As depicted in Figure 3, mouse anti-goat IgG was spotted on the first row of membrane at serial of concentrations (1, 0.1, 0.01 mg/mL) and goat anti-mouse IgG was spotted on the second row as a control at the same concentrations. Goat anti-mouse IgG functionalized AuNPs were prepared and spotted on the membrane. One can easily see the presence of the goat anti-mouse IgG-AuNPs on the mouse anti-goat IgG spotted strips as compared with the control goat anti-mouse IgG strips, demonstrating the specific recognition of the AuNPs (Figure 3a). Additionally, there is a concentration dependent positive signal in PEG2K@2S1L and the signals were significantly enhanced with the increasing ratio of the low molecular weight PEG on AuNPs as in PEG2K@4S1L. However, there was only negligible binding of the AuNPs functionalized with 5k PEG (Figure 3b), which is in good agreement with the results from the UV-Vis and DLS characterization. Severe non-specific adsorption only occurred in the case of AuNPs functionalized with low molecular weight PEG, as shown in Figure 3c (high binding on the control goat anti-mouse IgG strips). This is likely due to the severe particle aggregation after antibody functionalization as can also be seen from the broadening of UV-Vis spectra (PEG@anti-mouse IgG, Figure 2). In fact, aggregation was observed during the carbodiimide activation step as shown by change in the color of the solution. This phenomenon is consistent with our previous finding [29]. Similar results were obtained for larger AuNPs. Based on the physicochemical characterization with UV-Vis and DLS and the data obtained from the dot-blot immunoassay, PEG2K@4S1L was selected as optimal PEGylation and employed subsequently for the systematic investigation of the role of AuNP size in SP-LS.

### 3.4. Confirmation of Immuno-Binding Efficiency Using SPR

**Figure 4:**
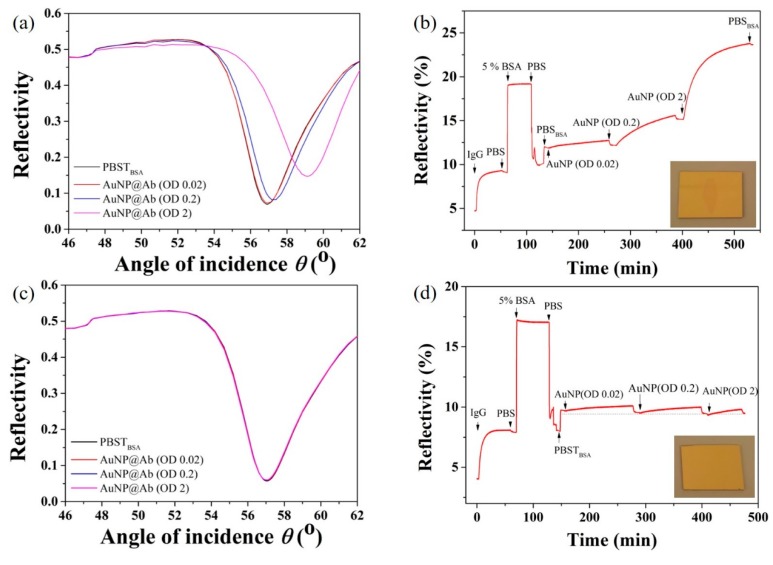
Validation of immune-binding assay for goat anti-mouse IgG functionalized 17 nm AuNPs on mouse anti-goat IgG SPR sensors. (**a**) Typical angular measurement as a function of AuNP concentration and (**b**) corresponding kinetic measurements; Control angular (**c**) and kinetic (**d**) measurements on goat anti-mouse IgG sensors as a function of AuNP concentration.

To further investigate the specificity and sensitivity of the biofunctionalized AuNPs in standard SPR refractometric biosensing, 17 nm AuNPs were PEGylated and conjugated with goat anti-mouse IgG antibody as described above. Mouse anti-goat IgG functionalized SPR sensors were exposed to goat anti-mouse IgG modified AuNPs of increasing concentrations and both the reflectivity versus angular shift and kinetic measurement were collected as shown in Figure 4a–d. As expected, the increment in angular shifts and changes in reflectivity were AuNP concentration dependent. Notably, there was a 0.4% reflectivity change, even at highly diluted AuNP concentrations, confirming the high binding ability of the Au tags on the biofunctionalized sensor surface which can be credited to the optimized bioconjugation. We also performed control experiments to evaluate the specificity of the biofunctionalized AuNPs. When goat anti-mouse IgG functionalized AuNPs were flowed over goat anti-mouse IgG immobilized SPR sensor surface, no significant AuNPs adsorption was measured both in angular and kinetic measurements. In addition, the specific adsorption of AuNPs on the SPR sensing area can be easily observed by the naked eyes at high AuNP concentrations (inserts, Figure 4b,d).

### 3.5. AuNP Size-Dependence in SP-LS Sensing

**Figure 5:**
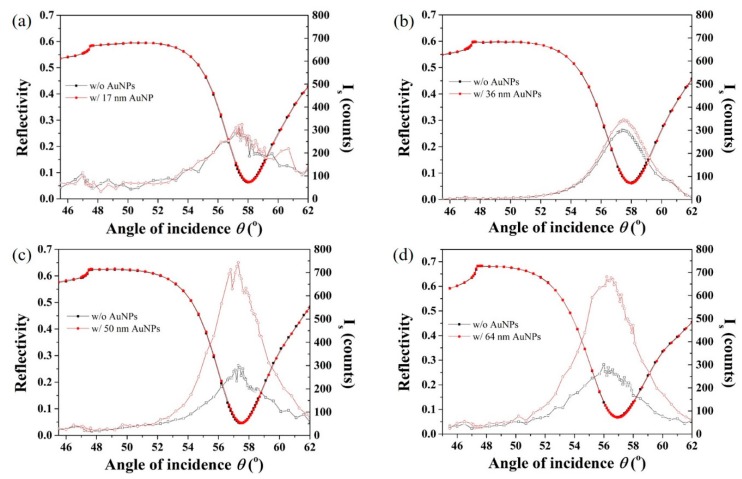
Angular reflectivity and scattering spectra as a function of the angle of incidence with or without the specific adsorption of AuNPs with diameters of (**a**) 17 nm, (**b**) 36 nm, (**c**) 50 nm and (**d**) 64 nm.

In our previous work, we have shown that 36 nm AuNPs used as SPR signal amplification tag yield approximately three orders of sensitivity enhancement in SP-LS sensing over conventional refractometric SPR. Simulation experiments shown in Figure 1 indicate that larger AuNPs results in higher scattered power in the SP-LS SPR sensing scheme. To experimentally verify this prediction, AuNPs with diameters of 17, 36, 50, and 64 nm were synthesized and modified with PEG2K@4S1L as described above and subsequently conjugated with goat anti-mouse IgG to investigate signal enhancement in SP-LS. However, larger AuNPs could not be acceptably bioconjugated as aggregation and sedimentation was observed for all the coatings tested. The SPR sensor surfaces were first modified with a mouse anti-goat antibody as for the previous refractometric SPR study. Typical angular reflectivity and scattering spectra are shown in Figure 5. Larger AuNPs with diameters of 50 nm and 64 nm induced significantly higher scattered light as compared with smaller AuNPs (17 nm and 36 nm), which is in agreement with the simulation results shown in Figure 1c. The AuNP tags concentration used in this experiment was very low (normalized to an optical density OD = 0.1) as higher concentrations resulted in signal saturation owing to the high sensitivity of the SP-LS sensing scheme. As a result, no angular shifts were measured in the measurements presented in Figure 5. No significant increase was measured for the 64 nm AuNPs in comparison to the 50 nm ones, which might be explained by the presence of small extend of aggregation in this sample.

Further optimization of the bioconjugation strategy is warranted to fully take advantage of the huge signal increase in the SP-LS scheme for very large AuNPs. However, in order to harness the potential of large AuNPs as a signal amplification tag, we investigated the feasibility of a two-step process referred to as AuNP enlargement mediated SP-LS. Gold and silver enhancement procedures have been widely employed for the enlargement of AuNP tags in immunoassays. In this work, PEGylated AuNPs with diameters of 17 to 64 nm were first enlarged with GE reagents in solution and characterized by SEM to monitor the resulting size increase.

**Figure 6:**
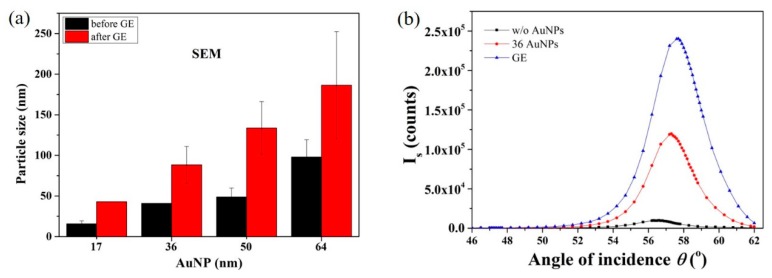
(**a**) Increases in diameters as determined by SEM for AuNPs of various size (17–64 nm) after a 5 min gold enhancement step; (**b**) SP-LS measurements before and after GE enlargement of 36 nm AuNP probes.

As shown in Figure 6a, the size of the AuNP increased to 43 nm, 88 nm, 134 nm and 186 nm, respectively for the 17, 36, 50 and 64 nm AuNPs. AuNPs with diameters of 36 nm were therefore selected to demonstrate the feasibility of AuNP enlargement mediated SP-LS as the size after GE (~100 nm) is predicted to yield extremely high signal amplification in SP-LS as per the theoretical modeling in Figure 1c. 36 nm of AuNPs bioconjugated with goat anti-mouse IgG reacted with the mouse anti-goat IgG functionalized SPR sensors, which was followed by incubation with the GE solution for 5 min. The scattered signal as a function of the angle of incidence was monitored by SP-LS as shown in Figure 6b. Approximately one-fold scattered signal enhancement was achieved after GE confirming the successful enlargement of the 36 nm AuNPs on the surface. Control experiment indicated no significant signal changes in the absence of the AuNP tags as shown in Figure S3, supporting information. While the enhancement measured here is lower than that predicted by the simulation, it conceptually demonstrates the feasibility of AuNP enlargement mediated SP-LS.

## 4. Conclusions

Building on our previous report of the exquisite sensitivity afforded by SP-LS sensing scheme in sandwich immunoassay format, we have investigated the size-dependent effect of AuNP signal amplification tags in the SP-LS sensing scheme, from both theoretical and experimental standpoints. Simulation demonstrated the direct relationship between the NP size and scattered signals. AuNPs with sizes of 100–130 nm were predicted to induce extremely high scattered signal. We then experimentally optimized a coating procedure based on mixed PEG molecules to maximize biding to solid substrate, while preventing aggregation of the AuNPs. However, AuNPs larger than 64 nm could not be satisfactorily bioconjugated with monoclonal antibodies, preventing their application in the SP-LS experimental study. Using a model immunoassay, we confirm the AuNP size-dependence of the SP-LS scheme. AuNPs with diameters of 50 nm and 64 nm provided a significantly increased signal in comparison to 17 and 36 nm AuNPs. Finally, in order to fully harness the potential of large AuNPs (>64 nm) as signal amplification tags for SP-LS, this study shows that a two-step protocol based on the gold enlargement of the AuNPs following their binding to their molecular targets on the sensor surface can be implemented.

## Figures and Tables

**Table 1:** Hydrodynamic diameters of as synthesized AuNPs (17 nm), and PEGylated and anti-mouse IgG bioconjugated AuNPs by dynamic light scattering.

	17 nm AuNP	PEG2k_S2L1	PEG2k_S2L1_IgG	PEG2k_S4L1	PEG2k_S4L1_IgG	PEG5k_S2L1_IgG	PEG5k_S4L1_IgG
Size (nm)	18.1 ± 0.1	30.6 ± 0.1	34.9 ± 0.3	32.2 ± 0.1	51.1 ± 0.1	35.0 ± 0.4	34.6 ± 0.2
PEG layer	-	12.5	12.5	14.1	14.1	-	-
IgG layer	-	-	4.3	-	18.9	-	-

## Supplementary Materials

The following are available online at http://www.mdpi.com/1424-8220/19/2/323/s1, Figure S1: Optical setup of SP-LS in this study. Figure S2: Calculated spectra for (a) normalized extinction efficiency Q_ext_ and (b) scattering efficiency Q_sca_ for different sized AuNPs; Figure S3: Control experiment for goat anti-mouse IgG functionalized SPR sensor surface exposed to GE reagent for 5 min and then measured by SP-LS. No signal enhancement is observed.
