# Supplementary material for: Surface Plasmon Enhanced Light Scattering Biosensing: Size Dependence on the Gold Nanoparticle Tag
Source: Sensors (Basel). 2019 Jan 15;19(2):323. doi: 10.3390/s19020323 (PMC6359480; doi:10.3390/s19020323)
Supplement: Supplementary file 1 [file sensors-19-00323-s001.pdf]

Supporting Information

Surface Plasmon Enhanced Light Scattering Biosensing: Size Dependence on the Gold Nanoparticle Tag

Chih-Tsung Yang [1], Yi Xu [2,3], Mohammad Pourhassan-Moghaddam [4,5], Duy Phu Tran [1], Lin Wu [2], Xin Zhou [6] and Benjamin Thierry [1,*]

[1] Future Industries Institute and ARC Centre of Excellence in Convergent Bio and Nano Science and Technology, University of South Australia, Mawson Lakes Campus, Mawson Lakes, 5095, Australia; chih-tsung.yang@unisa.edu.au (C.-T.Y.); duy.tran@unisa.edu.au (D.T.)

[2] Electronics and Photonics Department, Institute of High Performance Computing, Agency for Science, Technology, and Research (A*STAR) 138632, Singapore; yi_xu@mymail.sutd.edu.sg (Y.X.); wul@ihpc.a-star.edu.sg (L.W.)

[3] SUTD-MIT International Design Center & Science and Math Cluster, Singapore University of Technology and Design, 487372, Singapore

[4] School of Biomedical Engineering, University of Technology Sydney, Sydney, 2007, Australia; pourhassanmo@gmail.com

[5] Institute for Biomedical Materials and Devices (IBMD), Faculty of Science, University of Technology Sydney, Sydney, NSW, 2007, Australia

[6] Institute of Comparative Medicine, Yangzhou University, Yangzhou 225009, China; zhou_xin@126.com

* Correspondence: benjamin.thierry@unisa.edu.au

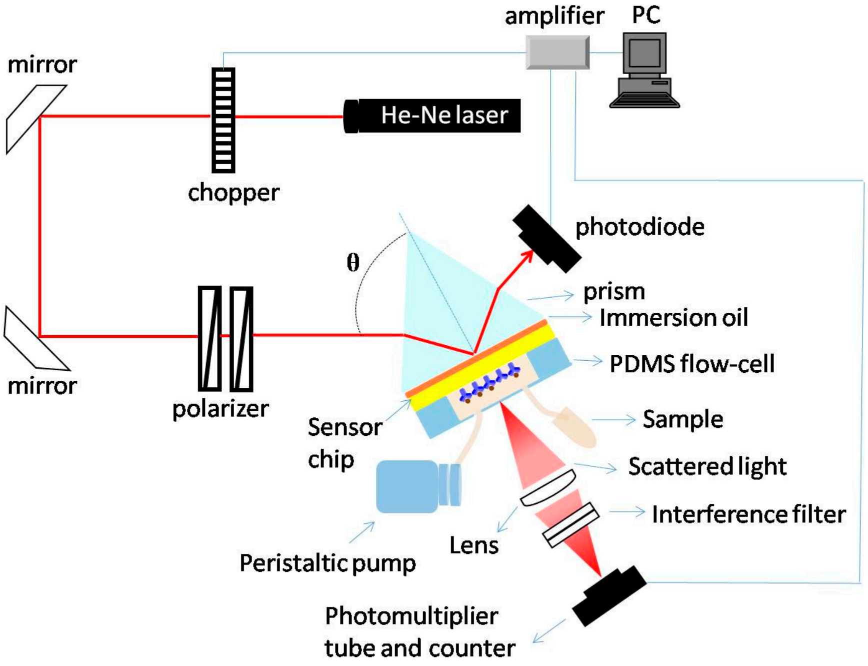

Figure S1. Optical setup of SP-LS in this study.

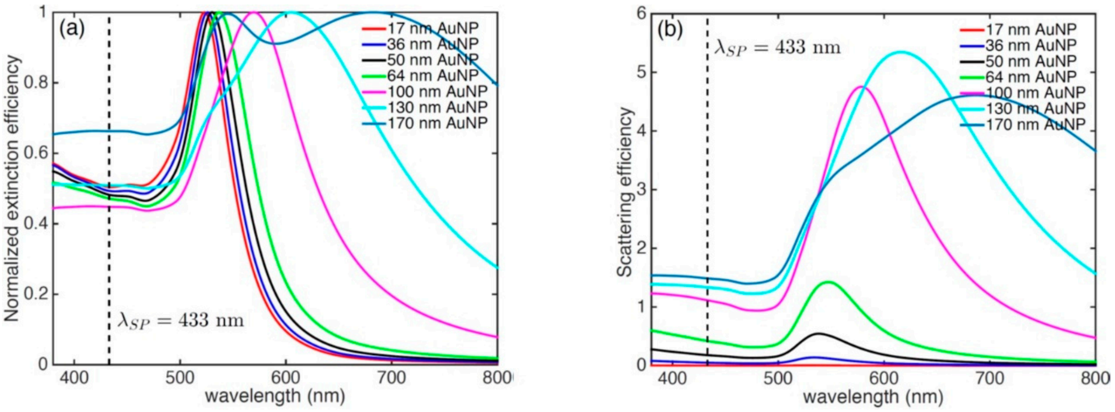

Figure S2. Calculated spectra for (**a**) normalized extinction efficiency Q_{ext} and (**b**) scattering efficiency Q_{sca} for different sized AuNPs.

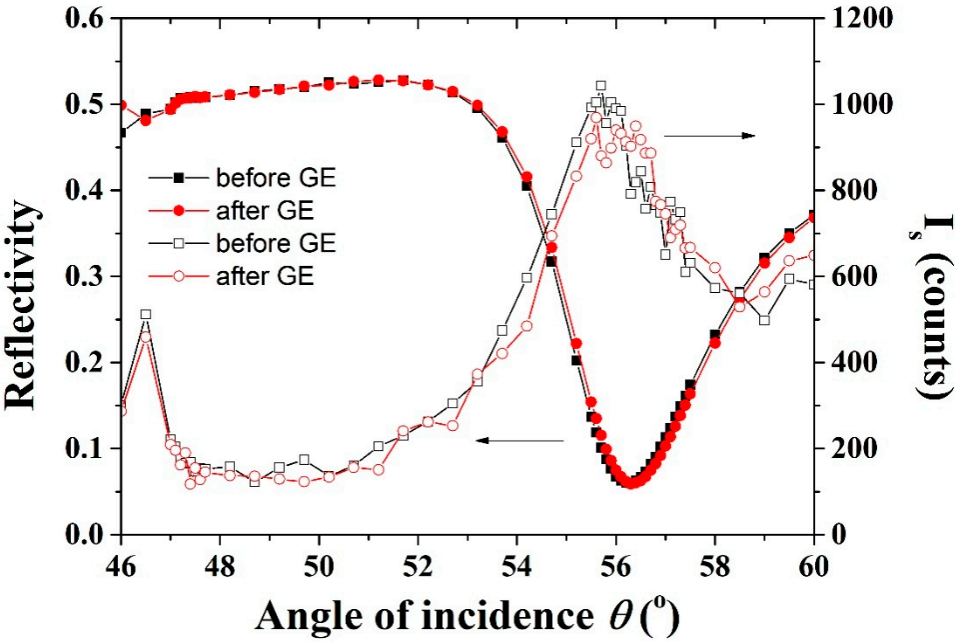

Figure S3. Control experiment for goat anti-mouse IgG functionalized SPR sensor surface exposed to GE reagent for 5 min and then measured by SP-LS. No signal enhancement is observed.
